# Supplementary material for: Causal Relationship between Aging and Anorexia Nervosa: A White-Matter-Microstructure-Mediated Mendelian Randomization Analysis
Source: Biomedicines. 2024 Aug 16;12(8):1874. doi: 10.3390/biomedicines12081874 (PMC11351342; doi:10.3390/biomedicines12081874)
Supplement: Supplementary file 1 [file biomedicines-12-01874-s001.zip › Supplementary Figure/Figure S8/Leaveoneout plot of MR analysis for TL to ubm-b-2066.pdf]

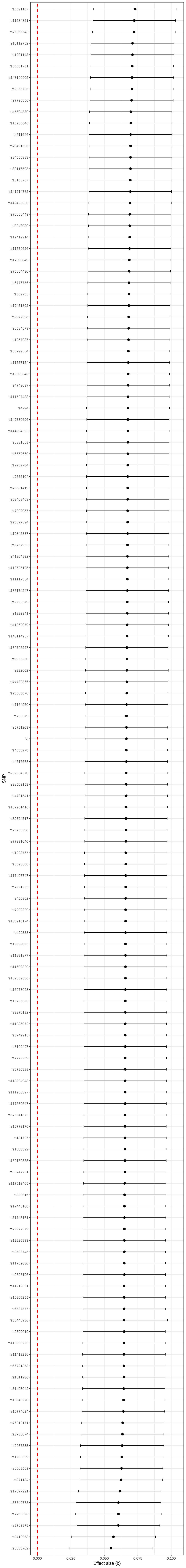

rs3891167
rs11584821
rs76065543
rs10112752
rs1291143
rs56061761
rs143190905
rs2056726
rs7790856
rs45604339
rs13230646
rs611646
rs78491606
rs34550383
rs80116508
rs8105767
rs141214782
rs142426306
rs76666449
rs9940099
rs12412214
rs11579626
rs17803849
rs75664430
rs6776756
rs869785
rs12451892
rs2977608
rs6584579
rs1957937
rs56799554
rs11557154
rs10805346
rs4743037
rs111527438
rs4724
rs142730696
rs144204502
rs6881568
rs6659669
rs2282764
rs2555104
rs73581419
rs59409453
rs7209057
rs28577594
rs10845387
rs3767952
rs41304632
rs113525195
rs11117354
rs185174247
rs2293579
rs1332941
rs41269079
rs145114957
rs139795227
rs9955360
rs932002
rs77732866
rs28363070
rs7164950
rs762679
rs6751209
All
rs4530278
rs4616688
rs202034370
rs28502153
rs4731541
rs137901416
rs80324517
rs73730598
rs77231040
rs1023767
rs3093888
rs117407747
rs7221585
rs450962
rs7099229
rs188918174
rs429358
rs13062095
rs11991877
rs11699829
rs182059586
rs16978028
rs10768683
rs2276182
rs11085072
rs5742915
rs8102497
rs7777289
rs6790988
rs112394943
rs111950327
rs117630647
rs376641875
rs10773176
rs131797
rs1003322
rs150150565
rs55747751
rs117512405
rs939916
rs17445108
rs61748181
rs79977579
rs12925933
rs2538745
rs11769630
rs9398196
rs11212631
rs10905255
rs6587577
rs35446936
rs9600019
rs116863223
rs11412296
rs66731853
rs1611236
rs61405042
rs10840270
rs10774624
rs76219171
rs3785074
rs2967355
rs1985369
rs6669563
rs871134
rs17677991
rs35640778
rs7705526
rs2763979
rs9419958
rs6536702
SNP
0.000
0.025
0.050
0.075
0.100
Effect size (b)
